# Real-Time GNSS-Based Attitude Determination in the Measurement Domain

**DOI:** 10.3390/s17020296

**Published:** 2017-02-05

**Authors:** Lin Zhao, Na Li, Liang Li, Yi Zhang, Chun Cheng

**Affiliations:** 1 College of Automation, Harbin Engineering University, Harbin 150001, China; zhaolin@hrbeu.edu.cn (L.Z.); lina@hrbeu.edu.cn (N.L.); zhangyi@hrbeu.edu.cn (Y.Z.); chengchun2@hrbeu.edu.cn (C.C.); 2 Academy of Opto-electronics, Chinese Academy of Sciences, Beijing 100094, China

**Keywords:** attitude determination, partial ambiguity resolution, measurement domain, real-time kinematic, GNSS

## Abstract

A multi-antenna-based GNSS receiver is capable of providing high-precision and drift-free attitude solution. Carrier phase measurements need be utilized to achieve high-precision attitude. The traditional attitude determination methods in the measurement domain and the position domain resolve the attitude and the ambiguity sequentially. The redundant measurements from multiple baselines have not been fully utilized to enhance the reliability of attitude determination. A multi-baseline-based attitude determination method in the measurement domain is proposed to estimate the attitude parameters and the ambiguity simultaneously. Meanwhile, the redundancy of attitude resolution has also been increased so that the reliability of ambiguity resolution and attitude determination can be enhanced. Moreover, in order to further improve the reliability of attitude determination, we propose a partial ambiguity resolution method based on the proposed attitude determination model. The static and kinematic experiments were conducted to verify the performance of the proposed method. When compared with the traditional attitude determination methods, the static experimental results show that the proposed method can improve the accuracy by at least 0.03° and enhance the continuity by 18%, at most. The kinematic result has shown that the proposed method can obtain an optimal balance between accuracy and reliability performance.

## 1. Introduction

Attitude determination (AD), which is always described with three parameters, e.g., yaw, roll, and pitch, is indispensable for many navigation systems. Compared with the other widely-used attitude sensors, such as inertial sensors and star trackers, the AD system based on GNSS (global navigation satellite system) has the advantages of drift-free, minor maintenance and relatively low-cost [1,2,3]. Therefore, the GNSS-based AD has drawn attention from the navigation community [4,5,6,7].

The GNSS based attitude parameters can be determined by using single or multiple antennas. The single-antenna GNSS-based system can estimate attitude by using the signal strength measurements [8] or the GNSS-derived acceleration [9,10]. A single antenna method is suitable for smart platforms such as pico- and nano-satellites, which require low-volume, low-complexity on-board systems. However, in order to obtain three attitude parameters, the single antenna-based AD needs auxiliary information, e.g., accelerometer or magnetometer measurements. Furthermore, the attitude accuracy of the single-antenna method ranges from 0.5° to 10° [8,9,10,11]. In contrast, the multi-antenna method can achieve attitude accuracy level at the order of 0.1° [3,4,5,12]. The multi-antenna GNSS-based system estimates the attitude by two or more antennas mounted on a platform. The accuracy and the reliability of AD is affected by at least three factors, i.e., the antenna configuration [13,14], the observation quality [15], and the attitude resolution method [16,17,18]. Generally, the antenna configuration is restricted by the geometry structure of the platform. Thus, in this contribution, we focus on the utilization of carrier-phase sufficiently to improve performance of the AD method.

At present, the multiple GNSS antennas-based AD method can be divided generally into two groups.

The first group includes the implementations in the position domain. These methods use the baseline vector to calculate the attitude parameters from the body fixed frame (BFF) to the reference frame. There are two widely-used position domain-based AD methods. One method is to estimate the attitude parameters by constructing a cost function of the baseline vector [19,20,21,22], which is the classic Wahba’s problem. This method is based on the singular value decomposition (SVD) to solve the attitude parameters and requires the non-coplanar baseline configuration. The other method is to estimate the attitude parameters by using the least square (LS) method [14,23], which can release the limitation for baseline configuration. However, these methods based on position domain resolve the baseline and the attitude parameters sequentially. The correlation between baselines is disregarded, which could undermine the reliability performance of AD. Furthermore, each baseline is estimated separately, and the redundancy for AD is inevitably reduced.

The second group is carried out in the measurement domain. According to the transformation relationships of the baseline vector from the BFF to the reference frame, the attitude parameters are introduced into the carrier phase observation model so that the attitude parameters can be solved directly [24,25,26,27,28]. Compared with the position domain-based AD methods, the measurement domain-based AD methods can determine the attitude parameters directly based on the ambiguity resolution (AR). The correlation between the attitude resolution and ambiguity resolution is, however, disregarded, which yields the reliability reduction of AD. Teunissen proposed a method to solve the attitude matrix and the ambiguity simultaneously to improve the solution performance [15,29]. Therefore, in this contribution, we propose a multi-antenna AD method in the measurement domain to implement the three independent attitude parameters and ambiguity resolution simultaneously, so that the accuracy and reliability of attitude solution can be improved.

The traditional GNSS-based AD methods use the high precision carrier phase measurements. The achievement of the high-precision of GNSS carrier phase measurements depends on whether or not the ambiguity can be correctly resolved. Therefore, the high-precision attitude resolution relies on the reliability of AR. When multi-frequency observations are used, the observation redundancy will be increased. The accuracy and reliability of AD can be improved by solving full ambiguity vectors. However, because of potentially severe observation noise and/or biases, it is difficult to fix a full ambiguity vector with a sufficiently low probability of false fixing [30,31]. If the ambiguity is biased because of low-quality carrier phase measurements, the accuracy of attitude solution will be jeopardized inevitably. Furthermore, the engaging of multiple constellations means that the real-time performance suffers from heavy computation burden because there has been a dramatic increase in available satellites. To fix these problems, a subset of decorrelated float ambiguities can be preferentially fixed so that the high-quality carrier phase measurements can be ultimately used without sacrificing the real-time performance [32,33]. Therefore, the partial ambiguity resolution (PAR) method can be used to satisfy the real-time and reliability requirements simultaneously.

In order to achieve the highest AD accuracy and reliability, firstly, the AD method is developed based on a multi-antenna redundant configuration in the measurement domain. To ultimately improve the AR reliability, the PAR method is applied based on minimum success rate criterion. Compared with the traditional AD methods, the proposed method can not only increase the redundancy of the AD model but also improve the AR reliability. Therefore, it can be predicted to achieve better AD performance.

The remainder of this paper is organized as follows: the next section derives the multi-antenna-based attitude resolution model in the measurement domain at on-the-fly mode. This is followed by a description of the attitude-ambiguity determination method based on PAR. We present and analyze the real-world static and kinematic experiments and summarize the research findings.

## 2. GNSS Attitude Resolution Model in the Measurement Domain

For GNSS-based AD systems, the attitude parameters can be estimated by using at least two non-collinear baselines. Carrier phase measurements must be adopted to achieve high-precision GNSS-based AD. In the traditional multi-epoch-based approaches, cycle slips must be detected and repaired, which is negative for the real-time applications. The single-epoch-based AD method in the measurement domain is, therefore, introduced so that the effect of cycle slips on the real-time operations can be avoided.

The baseline length is usually ultra-short at the meter level, or even the decimeter level. In such cases, the double difference (DD) processing is able to eliminate the common errors in DD observables, such as ephemeris error, atmospheric delay, and receiver clock error. Assuming the baseline *k* track *s* + 1 satellites on *f* frequencies, taking the satellite 1 as the reference, we cast the 2*fs* DD observation equations [34]:

(1) $$\begin{array}{l} E(\phi_{k})=(e_{f}⊗S_{k})x_{k}+(\Lambda ⊗I_{s})z_{k}\\ E(\rho_{k})=(e_{f}⊗S_{k})x_{k} \end{array}$$

where E(⋅) denotes the expectation operator, ⊗ denotes the Kronecker product, $\phi_{k}=[\phi_{k,1}^{T},\cdots ,\phi_{k,f}^{T}{]}^{T}$, $\rho_{k}=[\rho_{k,1}^{T},\cdots ,\rho_{k,f}^{T}{]}^{T}$, with $\phi_{k,n}$ and $\rho_{k,n}$ is the *s* × 1 DD carrier phase and code measurements on *n*th frequency; $x_{k}$ is the 3 × 1 unknown baseline vector in ECEF (Earth-centered Earth-fixed) frame and $x_{k}\in R^{3}$; $z_{k}=[z_{k,1}^{T},\cdots ,z_{k,f}^{T}{]}^{T}$ with $z_{k,n}$ is the *s* × 1 DD integer ambiguity vector on the *n*th frequency, and $z_{k}\in Z^{fs}$; $S_{k}={\left[{\left(-s_{k}^{12}\right)}^{T},\cdots ,{\left(-s_{k}^{1(s+1)}\right)}^{T}\right]}^{T}$ with $s_{k}^{\mathrm{ij}}$ is the 1 × 3 difference between the unit line-of-sight vector from baseline *k* to satellite pair *I* − *j*; $\Lambda =\mathrm{diag}(\lambda_{1},\cdots ,\lambda_{f})$ with $\lambda_{n}$ is the wavelength on *n*th frequency; $e_{f}={\left[1,\cdots ,1\right]}^{T}$ is the *f* × 1 unit vector; $I_{s}=\mathrm{diag}(1,\cdots ,1)$ is the *s* × *s* unit matrix.

The baseline *k* coordinate in BFF is denoted by $b_{k}$. The reference frame is usually a local level frame, such as the ENU (east-north-up) frame. Thus, the baseline vector **x***_k_* satisfies the coordinate transformation $x_{k}=C_{n}^{e}C_{b}^{n}b_{k}$, in which $C_{n}^{e}$ is the rotation matrix from the ENU frame to the ECEF frame, $C_{b}^{n}$ is the rotation matrix from the BFF to the ENU frame. The matrix $C_{b}^{n}$ contains the attitude parameters to be solved, which is also known as the attitude matrix.

Substitute $x_{k}=C_{n}^{e}C_{b}^{n}b_{k}$ relation into Equation (1), and combining the carrier phase and code measurements, the attitude equation expressed from Equation (1) as [15]:

(2) $$E(y_{k})=G_{k}C_{n}^{e}C_{b}^{n}b_{k}+F_{k}z_{k}$$

where $y_{k}$ is the 2*fs* × 1 DD observation vector with $y_{k}=[\phi_{k}^{T},\rho_{k}^{T}{]}^{T}$, $G_{k}$ is the 2*fs* × 3 geometry matrix with $G_{k}=e_{2}⊗e_{f}⊗S_{k}$, $F_{k}$ is the 2*fs* × 2*fs* design matrix with $F_{k}={\left[L^{T},0^{T}\right]}^{T}$ and $L=\Lambda ⊗I_{s}$.

In order to determine the attitude, the nonlinear Equation (2) is linearized around the attitude parameters. The attitude matrix $C_{b}^{n}$ can be divided into two parts, in which $C_{\hat{b}}^{n}$ is the rotation matrix from the estimated BFF to ENU frame, and $C_{b}^{\hat{b}}$ is the rotation matrix from the true BFF to the estimated BFF [24,35]. Given the estimation attitude **A**_0_ = [*θ*_0_ *φ*_0_ *ψ*_0_]^T^, in which *θ*, *φ*, *ψ* denotes the pitch, roll, and yaw angle, respectively, then:

(3) $$C_{b}^{n}(\theta ,\phi ,\psi )=C_{\hat{b}}^{n}(\theta_{0},\phi_{0},\psi_{0})C_{b}^{\hat{b}}(\delta \theta ,\delta \phi ,\delta \psi )$$

For the attitude error *δ***A** = [*δθ δφ δψ*]^T^, the matrix $C_{b}^{\hat{b}}$ can be approximately expressed as$C_{b}^{\hat{b}}(\delta \theta ,\delta \phi ,\delta \psi )\approx I_{3}+\delta A^{X}$, in which the *δ***A***^X^* is the cross product matrix for the vector *δ***A**. Thus, Equation (2) can be written as:

(4) $$\begin{array}{ll} E(y_{k}) & =G_{k}C_{n}^{e}C_{\hat{b}}^{n}(I_{3}+\delta A^{X})b_{k}+F_{k}z_{k}\\ & =G_{k}C_{n}^{e}C_{\hat{b}}^{n}b_{k}-G_{k}C_{n}^{e}C_{\hat{b}}^{n}b_{k}^{X}\delta A+F_{k}z_{k} \end{array}$$

This is equivalent to solving the model:

(5) $$E(\Delta y_{k})=H_{k}\delta A+F_{k}z_{k}$$

where $\Delta y_{k}=y_{k}-G_{k}C_{n}^{e}C_{\hat{b}}^{n}b_{k}$, $H_{k}=-G_{k}C_{n}^{e}C_{\hat{b}}^{n}b_{k}^{X}$.

Assuming that the number of antennas is *m* + 1 in the GNSS attitude system, we take antenna 1 as the reference antenna to constitute the *m* independent baseline vector. The single-epoch multi-frequency observation model is obtained as:

(6) E(y)=HδA+Fz

where $y={\left[\Delta y_{1}^{T},\cdots ,\Delta y_{m}^{T}\right]}^{T}$, $z={\left[z_{1}^{T},\cdots ,z_{m}^{T}\right]}^{T}$, $H={\left[H_{1}^{T},\cdots ,H_{m}^{T}\right]}^{T}$, $F=\mathrm{diag}(F_{1},\cdots ,F_{m})$. If *m* = 1, rank(**H**) = 2; else if *m* ≥ 2, rank(**H**) = 3. In order to determine the three independent attitude parameters, we need at least two non-collinear baselines for the proposed method.

For stochastic model of the observations **y** in Equation (6), we apply an elevation dependent weighting model [31,36], i.e., the variance of the un-differenced measurement *ξ* is $\sigma_{\xi }^{2}=a_{\xi }^{2}(1+1/{\left(\sin e\right)}^{2})$, with *e* the satellite elevation angle, $a_{\xi }$ related to the observation type. Suppose the un-differenced measurements used to construct the vector $y_{k}$ in Equation (2), are $y_{k,0}=[\phi_{k_{1},0}^{T},\rho_{k_{1},0}^{T},\phi_{k_{2},0}^{T},\rho_{k_{2},0}^{T}{]}^{T}$, with $\phi_{k_{n},0}$ and $\rho_{k_{n},0}$ as the carrier phase and code measurements from the receiver *k_i_* (*I* = 1, 2) to satellite *s* + 1 on frequency *f*, then, the variance-covariance(v-c) matrix of $y_{k,0}$ is by [37]:

(7) $$D(y_{k,0})=I_{2}⊗Q_{o}⊗I_{f}⊗Q_{e}$$

where D(⋅) denotes the dispersion operator, $Q_{o}$ and $Q_{e}$ are the observation and elevation dependent weightings, in which $Q_{o}=\mathrm{diag}(a_{\phi },a_{\rho })$, $Q_{e}=\mathrm{diag}(1+1/{\left(\sin e_{1}\right)}^{2},\cdots ,1+1/{\left(\sin e_{s+1}\right)}^{2})$. Assuming the observables are normally distributed and mutually uncorrelated, the v-c matrix of the DD observation vector y in Equation (6) can be expressed as:

(8) $$D(y)=Q_{\mathrm{yy}}=I_{m}⊗D(Ty_{k,0})$$

where $T=T_{1}^{T}⊗I_{2}⊗I_{f}⊗T_{s}^{T}$, with $T_{s}^{T}=[-e_{s},I_{s}]$ is the differencing matrix.

The GNSS observation model described in Equation (6) can be used to estimate the attitude and ambiguity parameters directly. It is noted the accuracy of AD can be determined better because a more accurate characterization on the correlation between the AD and AR has been described.

## 3. AD Based on PAR

The linear attitude observation described in Equation (6) can be solved by using the integer least square (ILS) theory. Firstly, the attitude estimate and the float ambiguity are calculated, then the fixed solution of AD can be obtained with the fixed ambiguity from the least squares ambiguity decorrelation adjustment (LAMBDA) method [38].

### 3.1. Float Solutions of AD

The float attitude-ambiguity solutions are estimated by Equation (6) using the least squares method, in which the integer nature of the ambiguities is disregarded. This solution is estimated as:

(9) $$\left[\begin{array}{c} \delta \hat{A}\\ \hat{z} \end{array}\right]={\left[\begin{array}{cc} H^{T}Q_{\mathrm{yy}}^{-1}H & H^{T}Q_{\mathrm{yy}}^{-1}F\\ F^{T}Q_{\mathrm{yy}}^{-1}H & F^{T}Q_{\mathrm{yy}}^{-1}F \end{array}\right]}^{-1}\left[\begin{array}{c} H^{T}\\ F^{T} \end{array}\right]Q_{\mathrm{yy}}^{-1}y$$

and the corresponding v-c matrix is expressed as:

(10) $$\left[\begin{array}{cc} Q_{\delta \hat{A}\delta \hat{A}} & Q_{\delta \hat{A},\hat{z}}\\ Q_{\hat{z},\delta \hat{A}} & Q_{\hat{z}\hat{z}} \end{array}\right]={\left[\begin{array}{cc} H^{T}Q_{\mathrm{yy}}^{-1}H & H^{T}Q_{\mathrm{yy}}^{-1}F\\ F^{T}Q_{\mathrm{yy}}^{-1}H & F^{T}Q_{\mathrm{yy}}^{-1}F \end{array}\right]}^{-1}$$

Compared with the ambiguity resolution for AD in position domain, the ambiguity resolution from Equation (6) can enhance the attitude precision and reliability by increasing the observable redundancy. Since the precision and the reliability of the ambiguity solution can be improved by more redundancy, the enhanced ambiguity performance can improve attitude accuracy and reliability. The proposed AD method from Equations (6) and (9) has more redundancy than the traditional position domain-based AD method. We can use a numerical example to explain the increased redundancy. If there are *m* baselines, the redundancy is *m* × (*s* × *f* − 3) for the traditional position-domain-based method, and, in contrast, there will be *m* × *s* × *f* − 3 redundancy for the proposed method. Since we need at least two non-collinear baselines for AD, thus, the proposed AD method can obtain more than 3 × (*m* − 1) redundancy than the traditional position-domain method.

### 3.2. Attitude and Ambiguity Fixing

One of the most important issues to obtain the GNSS-based precise attitude is to correctly solve the integer ambiguity. When using the multi-baseline and multi-frequency observables, the unknown ambiguity will increase so greatly that it is difficult to fix all the ambiguities reliably. Therefore, in this section we apply the PAR method, which improves the high-dimensional ambiguity reliability and the search efficiency of the LAMBDA method by fixing the subsets of ambiguities with higher priorities.

We can implement the PAR based on many criteria, e.g., elevation, signal-to-noise ratio, and minimum success rate requirement (MSRR) [31]. There will be a negative impact on the real-time performance if we consider all of the factors. Since the reliability of AR can be characterized by the success rate, the PAR method is, therefore, carried out based on the MSRR criterion.

The bootstrapped success rate has been proved to be a good approximation of the ILS success rate [39,40], which is used here. It can be computed by [41]:

(11) $$P_{s}=\prod_{i=1}^{n}\left(2\Phi \left(\frac{1}{2\sigma_{{\hat{z}}_{i|I}}}\right)-1\right)$$

where *n* is the number of ambiguities, $\sigma_{{\hat{z}}_{i|I}}$ is the *i*th ambiguity estimate conditioned on the previous I={i+1,⋯,n} sequentially rounded ambiguities, and the cumulative normal distribution as:

(12) $$\Phi (x)=\int_{-\infty }^{x}\frac{1}{\sqrt{2\pi }}e^{-\frac{v^{2}}{2}}dv$$

The overall success rate in Equation (11) is the product of the success rate for each individual ambiguity, the overall success rate reduced with fixing more ambiguities. After the ambiguity decorrelation process, all of the ambiguities are reordered in descending order according to their decorrelated variances, namely, the *n*th ambiguity has the smallest conditional variance. Thus, one starts with the *n*th decorrelated ambiguity, and checks if the success rate *P*_s_ is at least equal to the predefined MSRR (*P*_0_), until the largest possible subset K(*k*,…,*n*) is found. Then the ILS is applied to fix those *n* − *k* + 1 decorrelated ambiguities, the remaining *k* − 1 ambiguities are conditioned on the K(*k*,…,*n*) conditionally rounded ambiguities. In fact, the ILS-based success rate is equal to or higher than the bootstrapped success rate as calculated with Equation (11), since it is known that ILS is optimal [42].

The success rate can be determined before the actual integer ambiguities, and it is not directly dependent upon the actual measurements. However, any undetected biases for the measurements may influence on the real success rate, although the computed success rate could still be high. Acceptance of an incorrect integer will lead to an unacceptable attitude error, so the ratio test is added to ensure the reliability of the accepted integer ambiguities [43,44]. The test statistic for the ratio test is defined as the ratio of the minimum quadratic form of the residuals to the second minimum quadratic form of the residuals, i.e.,

(13) $$t=\frac{{\left\|\hat{z}-{\overset{⌣}{z}}_{O}\right\|}_{Q_{\hat{z}\hat{z}}^{-1}}}{{\left\|\hat{z}-{\overset{⌣}{z}}_{S}\right\|}_{Q_{\hat{z}\hat{z}}^{-1}}}$$

where ${\overset{⌣}{z}}_{O}$ and ${\overset{⌣}{z}}_{S}$ are the best and second-best ambiguity candidates. If *t* is less than or equal to a predefined threshold *μ*, the base ambiguity candidate is accepted. Otherwise, neither best nor the second-best can be accepted as the ambiguity fixed solution. Note that the ratio test is a member of the larger class of integer-aperture estimators and not optimal. The critical value *µ* determines the size of aperture of the pull-in region for the ratio test [45,46]. Since the reliability of ambiguity resolution is one of primary concerns for reliability of AD, a conservative critical value is, therefore, needed for the ambiguity validation.

Once the ambiguity vector obtained and validated correctly, the fixed solution of attitude parameter can determined as:

(14) $$\delta \overset{⌣}{A}=\delta \hat{A}-Q_{\delta \hat{A},\hat{z}}Q_{\hat{z}\hat{z}}^{-1}\hat{z}-\overset{⌣}{z})$$

The attitude solution from Equation (14) can be obtained with high accuracy and reliability by using the fixed ambiguity solution from $\overset{⌣}{z}$. Since the proposed MSRR-based PAR method can fix the ambiguities with sufficient confidence. Moreover, the ratio test is applied to assess if the best ambiguity solution should be accepted. Therefore, the proposed AD method is anticipated to obtain better ambiguity and attitude resolution performance.

## 4. Experiment and Analysis

In order to test the performance of the proposed AD method sufficiently, we conducted the static and the kinematic car experiments, respectively. The proposed measurement domain-based AD with partial ambiguity resolution (PAR-MAD) is compared with the position domain-based AD with full ambiguity resolution (FAR-PAD) and the measurement domain-based AD with full ambiguity resolution (FAR-MAD) by using the metrics of the accuracy, continuity, and reliability. The continuity is defined as the ratio between the number of ambiguity correctly fixed epochs and the number of total epochs. The reliability is characterized by the probabilities of false alarms and missed detections of the ambiguity resolution. The critical value *μ* for the ratio test is set up to 1/3 for the static and kinematic experiments, which is conservative for the reliability of AR. The FAR-MAD method is selected to demonstrate the effectiveness of PAR for AD. It is noted that the reference for the static experiment comes from the post-processing of commercial software in the ambiguity-fixed mode.

### 4.1. Static Experiment

**Figure 1:**
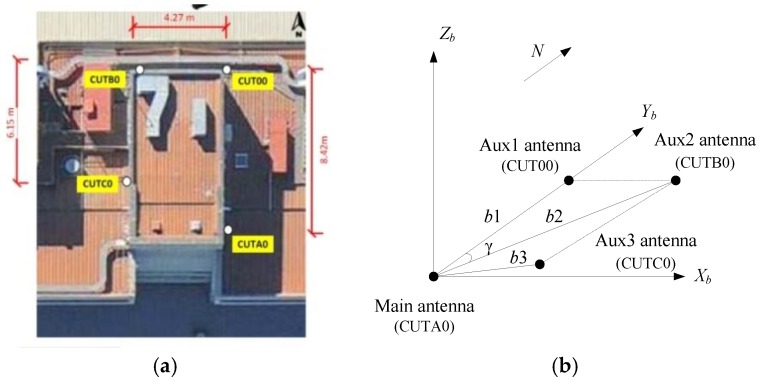
GNSS antenna used in static experiment: (**a**) is the antenna configuration; and (**b**) is the antenna geometry.

The static experimental data is downloaded from [47]. The data was collected by four TRM59800.00-SCIS antennas (Trimble Inc, Sunnyvale, CA, USA), which are connected with four TRIMBLE NETR9 receivers (Trimble Inc, Sunnyvale, CA, USA), on the roof of the Bentley campus building of Curtin University in Perth, Australia. The actual antenna configuration and the established BFF are shown in Figure 1, in which the antenna CUTA0 is selected as the master antenna, i.e., the origin of the BFF. The BFF in the static experiment is consistent with the ENU frame.

According to the provided true location of each antenna in the ECEF frame, we calculated their coordinates in the BFF and the corresponding baseline length, in which the antenna pair CUTA0-CUT00, CUTA0-CUTB0, CUTA0-CUTC0 form three baselines *b*1, *b*2, and *b*3, respectively, and the results are listed in Table 1. The reference attitude of the platform is determined as **A***_ref_* = (1.324, 177.1137, −0.0028)°, using the baselines *b*1 and *b*2.

**Figure 2:**
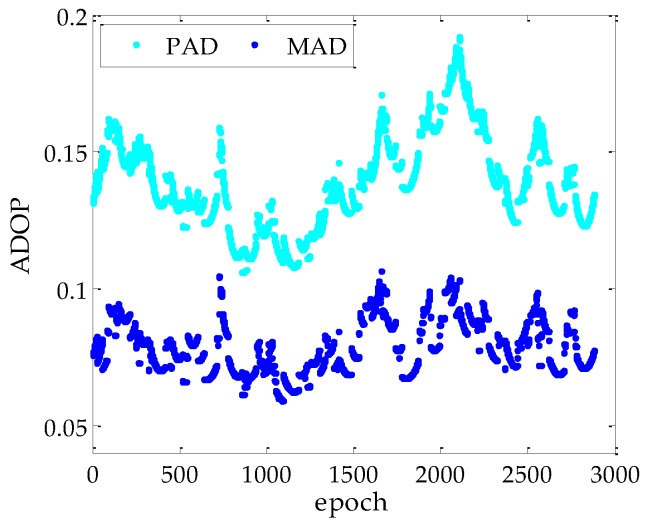
ADOP for the PAD and MAD methods.

In the static experiment, the GPS attitude system are formed by three baselines, the 24-h dual-frequency carrier phase and code data are used to obtain the single-epoch real-time solution. The data have a sample interval of 30 s. The cutoff satellite elevation angle is set to 5°. We select MSRR (*P*_0_) to rely on the ambiguity dilution of precision (ADOP) for the PAR method. Since the ILS success rate (*P_s_*_,*ILS*_) can be approximately expressed as a function depends on ADOP, the *P_s_*_,*ILS*_ will exceed 99% if ADOP is less than 0.15 cycles [30,31]. Figure 2 shows the ADOP obtained by the PAD and MAD methods, respectively. It can be observed that the ADOP for the MAD method is less than 0.1 cycles, which is remarkable better than the PAD. Therefore, the *P*_0_ is set to 99.9% in the static experiment. It can be anticipated that the MAD can achieve better success rate performance because of the increased redundancy.

**Figure 3:**
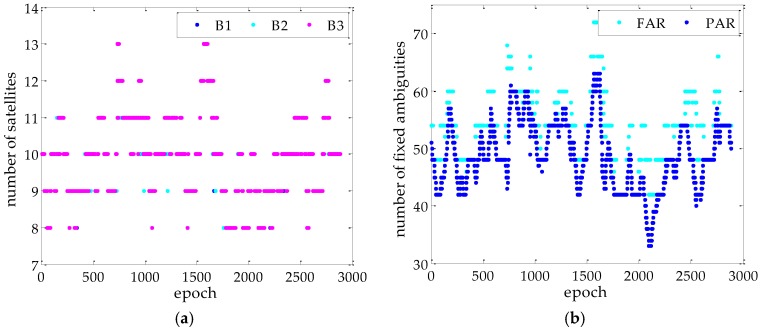
Visible satellite number for each baseline and fixed ambiguity number for FAR and PAR: (**a**) is the visible satellite number; and (**b**) is the fixed ambiguity number.

Figure 3 shows the number of the visible satellites and the fixed ambiguities for FAR and PAR based on MAD method. As shown in Figure 3, the each baseline is able to track 8–13 GPS satellites during the whole observation, and the fixed ambiguity for the PAR is less than that for the FAR. This implies that the success rate is lower than *P_0_* if the ambiguity is fixed with the FAR method. The 90th percentile of the fixed ambiguity component (*n*_fix_) is listed in Table 2.

**Figure 4:**
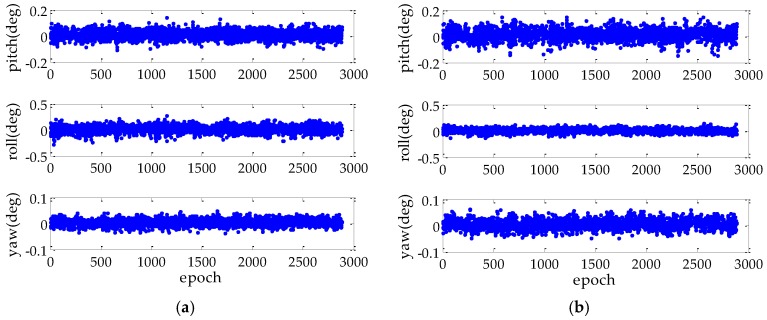

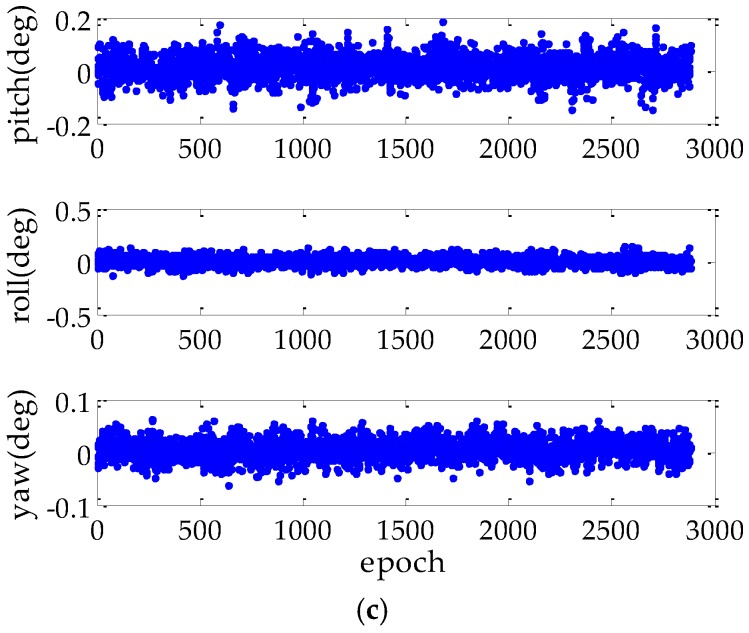
AD error comparison in static experiment: (**a**) is the FAR-PAD method; (**b**) is the FAR-MAD method; and (**c**) is the PAR-MAD method.

Figure 4 shows the attitude error for difference AD methods. It is obvious that the MAD methods can achieve better roll accuracy than the PAD methods, in contrast, the pitch and yaw error are comparable with these two methods. This is because the MAD method can use all of the measurements from each baseline. Furthermore, unlike the FAR-PAD resolving the attitude and ambiguity sequentially, the proposed PAR-MAD method resolves the attitude and ambiguity instantaneously with a more accurate characterization of the correlation between the AD and AR.

The statistical results of AR performance that include the probability of false alarm (*P*_fa_), the probability of missed detection (*P*_md_), and the AD accuracy and continuity (*P*_c_) for the above three AD methods are given in the Table 2. It should be noted that the *n*_fix_ for FAR-PAD is 20 for the single baseline. The results show that, when compared with the PAD method in the position domain, the proposed MAD method can decrease the roll standard deviation (STD) by 0.03°, while the pitch and yaw STD is increased by 0.008° and 0.013°, respectively, and also improves the spherical error probability (SEP 95) by 0.03°. Both MAD methods with the FAR and PAR can control the missed detection error because the observation redundancy is more than the PAD method. However, the FAR-MAD method has more false alarm errors. This is mainly because that the reliability of FAR is undermined by the low quality of carrier-phase measurements engaging in AR. As a result, although the FAR-MAD method has the highest attitude accuracy, the continuity of FAR-MAD is the worst. In contrast, the proposed PAR-MAD method can achieve a better false alarm and missed detection performance by selecting the subset of ambiguities and improve the continuity greatly by 18%. Therefore, it can be found that the proposed method can achieve the better reliability and continuity of AD.

### 4.2. Kinematic Experiment

**Figure 5:**
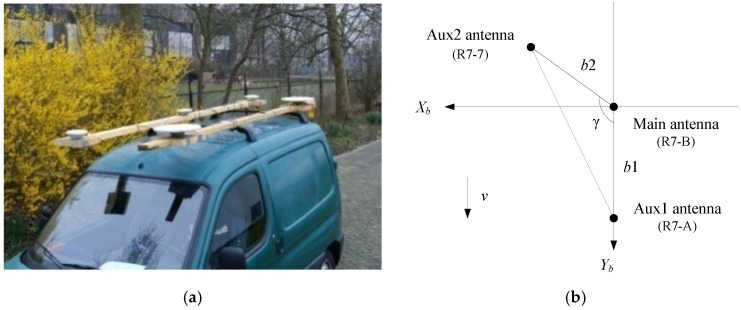
GNSS antennas configuration in kinematic experiment: (**a**) the antenna configuration; and (**b**) the antenna geometry indication.

The kinematic experiment was carried out on the multi-lane freeway, along Delft, in the Netherlands. The GNSS antennas are mounted on the test vehicle’s roof, as shown in Figure 5. To avoid the effect of the baseline distortion, two baselines are used to resolve the attitude angle, i.e., the three receivers, R7-A, R7-B, and R7-7 (Trimble Inc, Sunnyvale, CA, USA), are connected with the two Trimble Zephyr Geodetic antennas and a Trimble Zephyr antenna, respectively, of which the receiver R7-B corresponded to the master antenna defined as the origin of the BFF, and the receivers R7-A and R7-7 corresponded to the subjection antennas 1 and 2. The information of all the antennas in the BFF can be seen in Table 1. In the simulation, 6000 epochs of the GPS dual-frequency carrier phase and code measurements are used, and the car stays still in the former 2500 epochs. The kinematic data have a sample interval of 0.1 s. The cutoff satellite elevation angle is set to 5°. In this section, we compare the PAR-MAD method with the FAR-MAD method and FAR-PAD method using the two-baseline method, in which MSRR (*P*_0_) is set to 99.9%.

**Figure 6:**
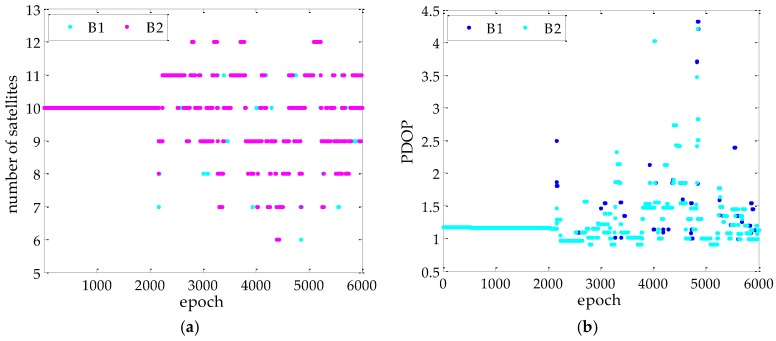
Visible satellite number and PDOP for the each baseline in kinematic experiment: (**a**) shows the visible satellite number; and (**b**) reflects the PDOP of PAD method.

Figure 6 shows the number of the visible satellite and the positional dilution of precision (PDOP) for each baseline in the kinematic test. After about 2100 epochs, the visible satellite number and the PDOP changed greatly. The visible satellite number varied from 6 to 12, and the PDOP can be larger than two, in which the maximum PDOP exceeded four. This means that the satellite geometry is poor during the experiment, which can be an indication of the extreme performance of different methods.

**Figure 7:**
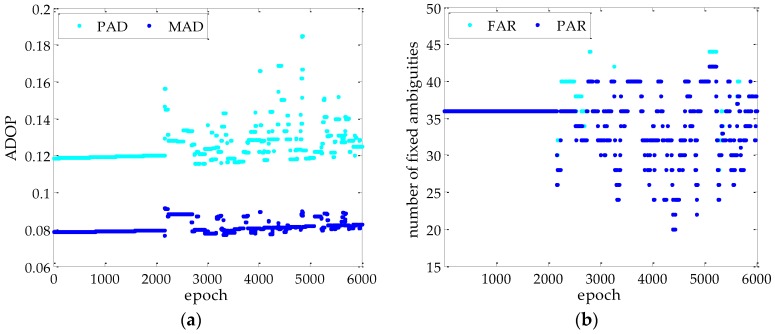
ADOP and fixed ambiguity number for different methods in kinematic experiment: (**a**) is the ADOP for the PAD and MAD methods; and (**b**) is the fixed ambiguity number of the MAD with FAR and PAR.

Figure 7 shows the ADOP for the FAR-PAD and PAR-MAD methods, and the fixed ambiguity number for the MAD methods with the FAR and PAR. The ADOP of the MAD is less than the PAD, which is about 2/3. Even in poor observation conditions, the ADOP for the MAD method is below 0.1 cycle. It follows that the ambiguities estimated by the MAD method have a higher accuracy. By comparing Figure 7a,b, it is obvious that when the ADOP is greater, the fixed ambiguity number with the PAR is less than the FAR. This means that the PAR method can guarantee the fixed ambiguity reliability by adjusting the fixed ambiguity subset, that the success rate is not less than MSRR, i.e., 99.9%.

**Figure 8:**
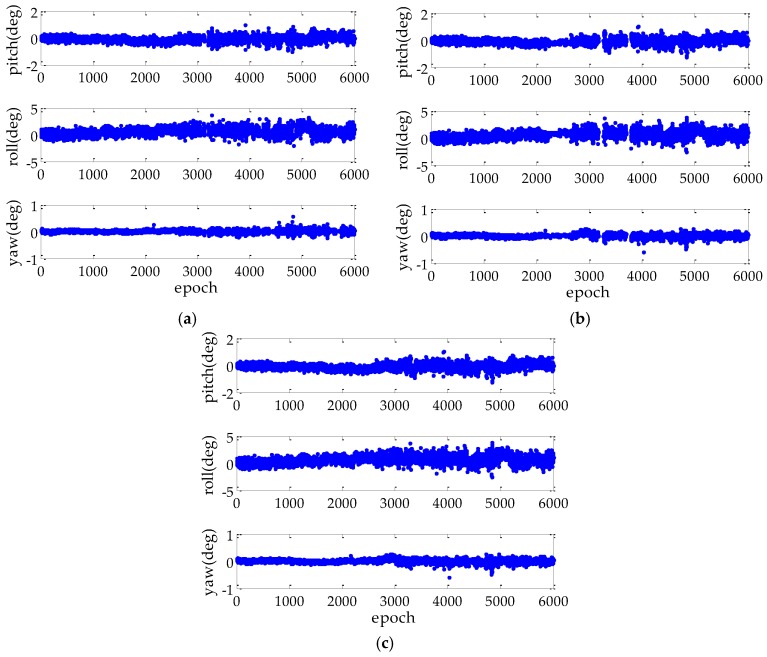
Attitude error for three AD methods in kinematic experiment: (**a**) is the FAR-PAD method; (**b**) is the FAR-MAD method; and (**c**) is the PAR-MAD method.

Figure 8 shows the attitude error for the FAR-PAD, FAR-MAD and PAR-MAD methods. For the attitude system formed by two baselines in kinematic experiment, the attitude error resolved by the three methods show comparable performance, of which the yaw error is the least, the pitch error takes second place, and the roll error is the greatest. In the figure we can also see that the proposed PAR-MAD method has better continuity. The statistical results of the AD performance comparison are listed in Table 3.

Table 3 lists the attitude accuracy, the continuity, and the false alarm and missed detection rates of the AR for different AD methods in the kinematic experiment. The statistical results show that the accuracy with the MAD method is slightly worse than the PAD method, regardless of the PAR-MAD and FAR-MAD methods. This is because the probability of a false alarm from the FAR-PAD method is much more than the FAR-MAD and PAR-MAD methods, respectively. These epochs with rejected ambiguities are excluded in the attitude accuracy evaluation. Furthermore, the statistics of AR shows that the PAR-MAD method has obtained higher reliability, which is reflected by the false alarm error decrement by at least 4%. Due to the increased AR reliability from the applied PAR method, the continuity for the PAR-MAD method improved by 11% and 1.1% relative to the FAR-MAD and FAR-PAD methods, respectively. The kinematic experiment result illustrates that the proposed AD method is able to balance the accuracy and reliability performance. In addition, compared with the static and kinematic estimation results in Table 2 and Table 3, the accuracy of AD is lower by about one order of magnitude in the kinematic experiment. It can be explained by the factors of the poorer navigation environment and the limited baseline configuration.

## 5. Conclusions

GNSS can be used to provide high-precision attitude information with the carrier phase measurements. In this paper, a real-time, high-accuracy, AD model is developed to satisfy the reliability requirement. The proposed method is tested by static and kinematic data and is compared with the traditional position domain-based methods. The static experiment demonstrates that the proposed AD method in the measurement domain (PAR-MAD) can obtain the best balance between the false alarm and the missed detection errors, when compared with the FAR-PAD and FAR-MAD methods. It can be further observed the best AD accuracy performance can be obtained from the proposed PAR-MAD. The kinematic result has also shown that the proposed PAR-MAD method can obtain an optimal balance between the accuracy and reliability performance. Both the static and kinematic experiments show that the proposed PAR-MAD method is effective for improving the reliability performance.

The better performance of the measurement domain-based method on the AD is achieved in two ways. Firstly, the AD resolution model is developed in the measurement domain to estimate the attitude and ambiguity simultaneously. The proposed method not only considers the correlation between the attitude and the ambiguity, but also makes full use of the multi-baseline measurements to improve the precision of unknown parameters. In addition, the reliability of AR and AD can be improved by using the MSRR-based PAR with the ratio test. Therefore, the proposed AD method in the measurement domain is more suitable for the applications which require higher reliability and continuity for AD, for example, for safety-critical applications.

## Figures and Tables

**Table 1:** GNSS baseline configuration in static and kinematic experiments.

Test Type	Baseline Coordinate in BFF (m)	Baseline Length (m)	Baseline Number
Static	*b*1 (0, 8.42, 0) *b*2 (4.27, 8.45, 0) *b*3 (5.23, 2.38, −0.19)	8.42 9.47 5.75	3
Kinematic	*b*1 (0, 2.20, 0) *b*2 (0.69, −0.29, 0)	2.20 0.75	2

**Table 2:** Statistical results of ambiguity resolution and AD for different methods in static experiment.

AD Method	STD (×10^−2^(°))			SEP 95 (°)	*P*_fa_ (%)	*P*_md_ (%)	*n*_fix_ (90%)	*P*_c_ (%)
	Pitch	Roll	Yaw					
FAR-PAD	2.93	6.34	1.22	0.1373	3.61	0.07	20	95.03
FAR-MAD	3.75	3.38	1.58	0.1010	19.86	0	60	80.00
PAR-MAD	4.23	3.84	1.78	0.1092	1.28	0	56	98.65

**Table 3:** Performance of ambiguity resolution and AD of three methods in the kinematic experiment.

AD Method	STD (°)			SEP 95 (°)	*P*_fa_ (%)	*P*_md_ (%)	*P*_c_ (%)
	Pitch	Roll	Yaw				
FAR-PAD	0.1850	0.6423	0.0471	1.6946	4.10	0	95.53
FAR-MAD	0.1887	0.6673	0.0579	1.7435	15.28	0	85.30
PAR-MAD	0.2049	0.6802	0.0611	1.8125	0.07	0	96.65
